# Alginate Particles for Encapsulation of Phenolic Extract from *Spirulina* sp. LEB-18: Physicochemical Characterization and Assessment of In Vitro Gastrointestinal Behavior

**DOI:** 10.3390/polym14214759

**Published:** 2022-11-06

**Authors:** Adriana R. Machado, Pedro M. P. Silva, António A. Vicente, Leonor A. Souza-Soares, Ana C. Pinheiro, Miguel A. Cerqueira

**Affiliations:** 1 School of Chemistry and Food, Federal University of Rio Grande, Rio Grande 96203-900, Brazil; 2 Centre of Biological Engineering (CEB), Campus de Gualtar, University of Minho, 4710-057 Braga, Portugal; 3 Associate Laboratory (LABBELS), Braga/Guimarães, Portugal; 4 International Iberian Nanotechnology Laboratory (INL), Av. Mestre José Veiga, 4715-330 Braga, Portugal

**Keywords:** antioxidant, biopolymers, controlled release, digestion

## Abstract

Encapsulation can be used as a strategy to protect and control the release of bioactive extracts. In this work, an extract from *Spirulina* sp. LEB-18, rich in phenolic compounds, was encapsulated in biopolymeric particles (i.e., composed of alginate) and characterized concerning their thermal behavior using differential scanning calorimetry (DSC), size, morphology, swelling index (S), and encapsulation efficiency (EE%); the release profile of the phenolic compounds at different pHs and the particle behavior under in vitro gastrointestinal digestion were also evaluated. It was shown that it is possible to encapsulate the phenolic extract from *Spirulina* sp. LEB-18 in alginate particles with high encapsulation efficiency (88.97%). It was also observed that the particles are amorphous and that the encapsulated phenolic compounds were released at a pH 7.2 but not at pH 1.5, which means that the alginate particles are able to protect the phenolic compounds from the harsh stomach conditions but lose their integrity under intestinal pH conditions. Regarding bioaccessibility, it was observed that the encapsulated phenolic compounds showed higher bioaccessibility compared to phenolic compounds in free form. This work increases the knowledge about the behavior of alginate particles encapsulating phenolic compounds during in vitro gastrointestinal digestion. It also provides essential information for designing biopolymeric particle formulations encapsulating phenolic compounds for application in pharmaceutical and food products.

## 1. Introduction

The incorporation of phenolic compounds (PC) into functional food products has several advantages as it allows preventing or delaying food spoilage, as well as acting as a functional compound with reported health benefits, giving rise to new and innovative functional foods; as such, it is a current focus of research in the food industry [1]. In order to be incorporated into functional food products, bioactive compounds, such as PC, need to maintain their stability, viability, and biological activity. As such, they need to be protected from pH, heat, moisture, and other extreme environmental conditions that lead to their degradation. Encapsulation techniques are among the most used solutions to provide needed protection for these compounds [2]. In fact, the main purpose of encapsulation is to protect a sensitive bioactive compound inside a particle or in its walls, physically isolating the compound from the external environment [3,4]. Encapsulation allows the development of formulations in which the bioactive compound is protected, and its release can be controlled with the intention of acting at a given location, for a set period of time and at a specific rate [5]. The ideal encapsulant for protection of bioactive compounds should have film-forming and emulsifying properties, be resistant to the gastrointestinal tract, have a low viscosity and a high solid content; it also should not be reactive with the encapsulated material and present a low hygroscopicity [6]. Among the materials commonly used for encapsulation, one class worth mentioning is represented by polysaccharides, such as sodium alginate, which is considered one of the most widely used. Sodium alginate is a polysaccharide derived from brown seaweeds (Phaeophyceae) that is commonly used as thickener, stabilizer, and gelling agent in the food and pharmaceutical industries [7]. Alginate consists of alternating blocks of 1–4 linked β-(D)-mannuronic (M) acid and α-(L)-guluronic acid (G). Due to the presence of carboxylic groups on both monomers, alginate has a negative charge above its pKa (3.3–3.5) [8,9]. Some of the excellent properties of alginate are biocompatibility, hydrophilicity, and biodegradability under normal physiological conditions [10]. Another important property of alginate is its ability to react with divalent cations, especially calcium ions, to produce strong gels or insoluble polymers. Alginate-based gels upon the binding of divalent ions of calcium to guluronic acid blocks in alginate chains, resulting in a structure named the “egg box” model [11]. Ionic gelation is an encapsulation technique that makes use of the opposite charge between a polymer and an ionic solution, e.g., alginate and calcium chloride, to create a crosslinked gel. When a bioactive compound is mixed with the sodium alginate solution and extruded through a syringe dropwise into a calcium solution, the developed crosslinked alginate structure takes the shape of alginate particles that are able to encapsulate the bioactive compound within their core [12,13]. As such, over the past years, the use of sodium alginate has increased in various technological fields such as food, pharmaceutical and agriculture to produce capsules, particles, and microbeads that are able to encapsulate and protect bioactive compounds [14,15,16]. The ionic gelation technique with alginate has been used for the encapsulation of several extracts such as anthocyanins from *Hibiscus sabdariffa* L. [17], calyces extract of *Stevia rebaudiana* Bertoni leaf [18], and extracts from annatto seeds [19], from Amazonian berries [20], and from *Viola odorata* Linn [21]. *Spirulina* sp. LEB-18 has remarkable potential to be explored in a sustainable manner as a source of bioactive compounds with high antioxidant activity (e.g., PC). This microalga is classified as GRAS (generally recognized as safe) by the FDA (Food and Drug Administration) and European Food Safety Authority (EFSA), which ensures its safety when used as a food ingredient [22,23,24]. Alginate microparticles can preserve or even enhance the antioxidant action of PC, by facilitating the penetration of phenolic acids through the cellular wall and membrane, allowing its action in metabolic processes and a controlled release of the PC to the desired medium, while avoiding their chemical degradation [25,26,27]. There are some studies addressing the encapsulation of phenolic extract of *Spirulina* in alginate particles. Rajmohan and Bellmer [28] produced *Spirulina*-loaded alginate particles using ionic gelation, and characterized them in terms of particle size, texture, morphology, and crude protein content of the particles, while other authors [29] assessed the sensory effect of microencapsulated *Spirulina* in fortified yoghurt, assessing microparticle size and morphology, *Spirulina* release from the alginate microparticles, and the sensory properties of the fortified yoghurt. In 2020, Zen et al. [30] produced a functional pasta with microencapsulated *Spirulina* and assessed its stability against heat treatment, as well as the sensory parameters of the developed pasta. Nevertheless, these studies involving the microencapsulation of *Spirulina* did not assess the protection and stability of *Spirulina* conferred by its encapsulation in alginate particles, i.e., when subjected to an in vitro gastrointestinal digestion process, nor did they assess the bioactive properties and bioaccessibility of encapsulated *Spirulina* after gastrointestinal digestion. Abraham et al. [31] assessed the behavior of microencapsulated *Spirulina* particles under gastrointestinal digestion but the study only focused on how the viscosity of alginate can affect the release profile of encapsulated bioactives under digestion. Furthermore, this work did not study the release profile of these bioactives in different media to understand the release mechanisms involved, before submitting the particles to in vitro digestion.

In this context, the objective of this work was to encapsulate the PC extracted from *Spirulina* microalgae-LEB-18 in alginate particles, characterize them, and evaluate their release behavior in media with different pH and when submitted to an in vitro gastrointestinal digestion, providing information regarding PC release, bioaccessibility, and bioactivity. This work is aimed at assessing if PC from microalgae incorporated into alginate particles can be a route to increase phenolic stability, antioxidant efficiency and shelf life of foods.

## 2. Materials and Methods

### 2.1. Raw Materials

*Spirulina* biomass (LEB strain-18), batch 2014, was provided by the Biochemical Engineering Laboratory of the Federal University of Rio Grande, isolated from the Mangueira lagoon (33°302′133 S, 53°082′593 O), located in Santa Vitória do Palmar, RS, Brazil [32] and supplemented with 20% Zarrouk medium [33]. The dried biomass was provided in the form of pellets and subsequently ground in a ball mill (Model Q298-2) and sieved in a sieve shaker, 200 mesh, to homogenize the particle size, resulting in a particle size of 88 µm. Powdered biomass was packaged in vacuum inside high-density polyethylene containers (HDPE) with a capacity of 500 g and stored under refrigeration at a temperature of ~7 °C until further analysis. Pepsin from porcine gastric mucosa (≥2500 U·mg^−1^), bile extract porcine, pancreatin from porcine pancreas (8× USP), Pefabloc^®^ SC, and the salts used for the preparation of oral, gastric, and intestinal electrolyte solutions were purchased from Sigma-Aldrich. Sodium alginate (PROTONAL CR8223, M/G ratio 65/35 and 259–340 kDa) from FMC Biopolymer was kindly supplied by Eurosalmo (Portugal). Calcium chloride (CaCl_2_) was purchased from Panreac (Barcelona, Spain).

### 2.2. Phenolic Compound Extraction

The extraction of the PC was performed according to Souza [34], with some modifications. Briefly, 2 g of dried microalgae were weighed, 25 mL of methanol was added to each sample, followed by agitation in an orbital shaker table TE-141 (TECNAL), at a temperature of 45 °C for 120 min at 230 rpm. Subsequently, centrifugation at 3220× *g* for 15 min was carried out, and the solvents were evaporated in a rotary evaporator at 50 °C. The extracts were filtered and clarified with 10 mL of 0.1 mol·L^−1^ barium hydroxide and 10 mL of 5% zinc sulfate solution. The solutions were filtered and transferred to a volumetric flask of 50 mL, completing the final volume with water.

### 2.3. Quantification of Total Phenolics

Quantification of total phenolic content in the extracts was carried out spectrophotometrically using the Folin–Ciocâlteu method according to Souza et al. [35]. Furthermore, 500 µL of extract was used, in addition to 500 μL of distilled water and 4.5 mL of alkaline solution (carbonate sodium 4% (*w*/*v*), copper sulfate 2% (*w*/*v*), and double tartrate of sodium and potassium 2% (*w*/*v*)). After homogenization, samples were subjected to heating at 40 °C for 15 min. After this time, 500 μL of 2 mol·L^−1^ Folin–Ciocâlteu reagent (diluted 1:2 with distilled water) was added. The absorbance of the samples was measured at 760 nm after 10 min of rest at room temperature. The total phenolic content of the extracts was determined by interpolating the absorbance of the sample against an analytical curve constructed with a standard solution of gallic acid with concentration levels from 5 to 35 μg/mL, and the results were expressed in mg of gallic acid (GAE)·g^−1^ of microalgae. The determination coefficient of the calibration curve was 0.9831, and the equation of the curve was *y* = 0.029*x* − 0.030 (µg·mL^−1^).

### 2.4. Encapsulation of Phenolic Compounds by Ionic Gelation

Particles were produced using an adaptation of the methodology proposed by other authors [36,37,38]. Briefly, alginate particles were produced using the ionic gelation technique. Firstly, different concentrations of alginate (2%—T1, 1.5%—T2, and 1%—T3) and calcium chloride (1.0, 0.5, and 0.25 mol·L^−1^) were prepared and mixed with the phenolic extract to produce alginate particles encapsulating the PC through ionic gelation. Using a syringe pump (NE-1002X Programmable Microfluidics), the alginate solution containing PC (2.55 mg gallic acid∙g^−1^ of microalgae), solubilized with ultrapure water, was extruded dropwise, under magnetic stirring at 300 rpm for 20 min, into a calcium chloride solution, and a rapid gelation of the solution occurred. After this process, the particles were kept under stirring at 600 rpm for 30 min. Finally, the particles were washed with deionized water and dried at room temperature (~21 °C) and their morphology, size, and encapsulation efficiency were evaluated.

### 2.5. Characterization of the Alginate Particles

#### 2.5.1. Optical Microscopy

Alginate particles were on in a coverslip, and a microscopic visualization of the particles was performed to assess their shape and size. In order to measure the size of the particles, an OLYMPUS magnifying glass (OLYMPUS SZ-CTV, Japan) was used. The samples were photographed with a magnification of 0.67 using the program “Image-Pro Plus” (op + I), and light position, contrast, and brightness values were also defined. The pictures were then opened, and calibration graph paper 0.67 was chosen [39]. To determine the average diameter of the particles, granulometric analysis was performed on approximately 25 particles.

#### 2.5.2. Encapsulation Efficiency (% EE)

Encapsulation efficiency was determined as follows: 0.5 mL of alginate particles containing the PC and 1 mL of 3% sodium citrate were added to an Eppendorf tube. After homogenization and centrifugation at 14,000× *g* for 30 min, two separate phases were obtained. The supernatant was removed and quantified for total phenolic content as presented in Section 2.3. The encapsulation efficiency (*EE*) was calculated as shown in Equation (1). This parameter was determined through the ratio between PC encapsulated and the total phenolics (*T_P_*). The quantification of encapsulated PC was determined by the difference between the total phenolics (*T_P_*) and the quantification of the free (i.e., non-encapsulated) phenolics (*F_P_*).

(1) $$EE\left(\%\right)=\left[\frac{\left(T_{P}-F_{P}\right)}{T_{P}}\right]\times 100.$$

#### 2.5.3. Swelling Index

Swelling index (*S*) measurements were performed by weighing on a precision balance (Model: SBS-LW-200A), 10 mg of dried particles with and without PC, and placing them in containers with distilled water, at 0, 30, 60, 90, and 120 min. Periodically, the particles were removed from the containers, the excess water was removed by placing them on a thin absorbent paper, and then the soaked particles were weighed [40,41]. *S* was obtained according to Equation (2).

(2) $$S=\frac{M_{t}-M_{o}}{M_{o}},\;$$

where *M*_0_ is the initial mass of the dry particles, and *M_t_* is the mass of the particles after immersion in water at a given time interval.

#### 2.5.4. Release Profile

The release profile of the PC was determined according to the procedure described by Barbizan [42]; Kleinubing [43], with some modifications. The release of PC of microalgae was evaluated by immersing the alginate particles in HCl at pH 1.5, and in PBS at pH 7.2, to simulate the pH of gastric and intestinal environments, respectively. Approximately 50 mg of particles were placed in the release medium and were maintained under stirring at 400 rpm rotation speed, with a temperature of 37 ± 0.5 °C. Aliquots were taken periodically. The concentration of PC was determined by measuring the absorbance at 760 nm in a spectrophotometer (Synergy HT (Bio-Tek, EUA). Readings were performed in triplicate.

In order to evaluate the release mechanism of PC from alginate particles, a kinetic model that accounts for both Fickian and Case II transport effects in hydrophilic matrices, the linear superimposition model (LSM), was applied [44]:

(3) $$M_{t}=M_{t,F}+M_{t,R},$$

where *M_t,F_* and *M_t,R_* are the contributions of the Fickian and relaxation processes, respectively, at time *t*. The purely Fickian process is described by

(4) $$M_{t,F}=M_{\infty ,F}\left[1-\frac{6}{\pi^{2}}\overset{\infty }{\underset{n=1}{\sum }}\frac{1}{n^{2}}\exp(-n^{2}k_{F}t)\right],$$

where $M_{\infty ,F}$ is the compound release at equilibrium, and *k_F_* is the Fickian diffusion rate constant. Equation (4) can be simplified using the first term of the Taylor series [45]. As for polymer relaxation, it is driven by the swelling ability of the polymer (i.e., alginate) and is then related to the dissipation of stress induced by the entry of the penetrant, which can be described as a distribution of relaxation times, each assuming a first order-type kinetic equation [44]:

(5) $$M_{t,R}=\underset{i}{\sum }M_{\infty ,R_{i}}\left[1-exp\left(-k_{R_{i}}t\right)\right],$$

where $M_{\infty ,R_{i}}$ is the contribution of the relaxation processes for compound release and $k_{R_{i}}$ is the relaxation rate constant. For most cases, there is only one main polymer relaxation that influences transport; thus, the above equation can be simplified using i=1.

Therefore, the linear superimposition model (LSM) for compound release from particles can be described by

(6) $$\frac{M_{t}}{M_{\infty }}=X\left[1-\frac{6}{\pi^{2}}exp\left(-k_{F}t\right)\right]+\left(1-X\right)\left[1-exp\left(-k_{R}t\right)\right],\;$$

where *X* is the fraction of compound released by Fickian transport.

The experimental results were analyzed by fitting Equation (6) to assess the transport mechanism involved in the release of PC from alginate particles.

#### 2.5.5. Differential Scanning Calorimetry—DSC

Calorimetric studies were conducted using a Perkin Elmer DSC 4000 differential scanning calorimeter (Perkin Elmer, Waltham, MA, USA). First, 10 mg of alginate particles were placed in aluminum DSC pans and sealed before analysis. Samples were analyzed from 20 to 250 °C at a heating rate of 30 °C/min under an argon atmosphere. A sealed empty pan was used as reference. Melting temperature peaks (*T_m_*), and the onset and ending temperatures were calculated using Pyris software version 11.1.

### 2.6. Behavior under In Vitro Gastrointestinal Digestion

#### 2.6.1. In Vitro Gastrointestinal Digestion

A harmonized static in vitro model was used to evaluate the digestibility of alginate particles containing PC. Mouth, stomach, and small intestinal steps were simulated using the protocol described by Minekus et al. [46]. Briefly, 5 mL of alginate particles containing PC were used in the tests. The oral phase simulation consisted of the addition of simulated salivary fluid (SSF) (KCl 15.1 mmol·L^−1^, KH_2_PO_4_ 3.7 mmol·L^−1^, NaHCO_3_ 13.6 mmol·L^−1^, MgCl_2_(H_2_O)_6_ 0.15 mmol·L^−1^, (NH_4_)_2_CO_3_ 0.06 mmol·L^−1^, CaCl_2_(H_2_O)_2_ 1.5 mmol·L^−1^, and HCl 1.1 mmol·L^−1^), CaCl_2_(H_2_O)_2_ (in order to achieve 0.15 mmol·L^−1^ in the fluid), and purified water. The mixture was incubated for 2 min at 37 °C. Note that amylase was not used in the oral phase as these samples did not contain starch. The gastric phase simulation consisted of porcine pepsin solution (2000 U/mL in the final mixture), simulated gastric fluid (SGF) (KCl 6.9 mol·L^−1^, KH_2_PO_4_ 0.9·mmol.L^−1^, NaHCO_3_ 25 mol·L^−1^, NaCl 47.2 mol·L^−1^, MgCl_2_(H_2_O)_6_ 0.1 mol·L^−1^, (NH_4_)_2_CO_3_ 0.5 mmol·L^−1^, CaCl_2_(H_2_O)_2_ 0.15 mol·L^−1^, and HCl 15.6 mmol·L^−1^), CaCl_2_(H_2_O)_2_ (in order to achieve 0.15 mmol·L^−1^ in the fluid), HCl to adjust the pH to 3.0, and purified water. Samples were incubated in a shaking bath at 37 °C for 2 h. The intestinal phase was simulated by adding simulated intestinal fluid (SIF) (KCl 6.8 mmol·L^−1^, KH_2_PO_4_ 0.8 mmol·L^−1^, NaHCO_3_ 85 mmol·L^−1^, NaCl 38.4 mmol·L^−1^, MgCl_2_(H_2_O)_6_ 0.33 mmol·L^−1^, CaCl_2_(H_2_O)_2_ 0.6 mmol·L^−1^, and HCl 8.4 mmol·L^−1^), CaCl_2_(H_2_O)_2_ (in order to achieve 0.3 mmol·L^−1^ in the mixture), pancreatin suspension in SIF (based on trypsin activity of 100 U/mL in the final mixture), bile solution in SIF (in order to reach the concentration of 10 mmol·L^−1^ in the final mixture), NaOH (volume necessary to adjust the pH to 7.0), and purified water. The samples were then incubated for 2 h, at 37 °C.

Samples were collected after each phase (oral, gastric, and intestinal) of the in vitro digestion and the reaction of the gastric phase (pepsin activity) was stopped by increasing the pH of that phase to 7.0 with NaHCO_3_ (1 mol·L^−1^) and after full digestion; the reaction was stopped by the addition of the enzyme inhibitor pefabloc (1 mmol·L^−1^) (10 µL for each 1 mL of sample). All samples were tested at least in triplicate.

#### 2.6.2. Quantification of Total Phenolic Compounds after In Vitro Gastrointestinal Digestion

Quantification of total phenolics content in the samples obtained after each stage of the in vitro gastrointestinal digestion (oral, gastric, and intestinal) was carried out using UV/Vis spectroscopy (VARIAN/CARY-100), and the Folin-Ciocalteu method according to Souza et al. [35], as described in Section 2.3.

#### 2.6.3. Antioxidant Activity of the Extracts after the In Vitro Gastrointestinal Digestion

The antioxidant activity was measured along the digestion process using the DPPH (2,2-diphenyl-1-picrylhydrazyl) antioxidant assay, to assess the antioxidant ability of free and encapsulated PC. The antioxidant activity of the encapsulated PC submitted to the gastrointestinal digestion was measured using the procedure described by Herrero et al. [47] with some modifications. The measurements were performed in a UV/Vis spectrophotometer (VARIAN/CARY-100) at a wavelength of 515 nm. To the tubes containing 3.0 mL of methanolic DPPH solution (5.2 × 10^−5^ mol/L^−1^), 0.5 mL of methanol and 0.5 mL of free or encapsulated PC subjected to the in vitro gastrointestinal system were added. The reactive mixture was held at room temperature shielded from the incidence of light, and the yellow to violet color change was measured after 30 min of reaction. The DPPH solution was prepared daily, stored in amber bottles covered with aluminum foil, and kept in the dark at 4 °C until measurements were performed.

The ability to sequester free radicals was expressed as a percentage of oxidation inhibition of the radicals and calculated according to Equation (7).

(7) $$Inhibition\left(\%\right)=\left(\frac{A_{Ext}-A_{DPPH}}{A_{DPPH}}\right)\times 100,\;$$

where *A_DPPH_* is the absorbance of the DPPH solution, and *A_Ex_*_t_ is the absorbance of the sample solution. *A_Ext_* was calculated as the difference between the absorbance of the test sample solution and its blank.

#### 2.6.4. Bioaccessibility

In order to determine bioaccessibility, it was assumed that the fraction of PC that ended up in the micelle phase was its bioaccessible fraction [48,49]. The bioaccessibility of free and encapsulated PC obtained from LEB-18 *Spirulina* was determined as previously described [48,49]. Samples (5 mL) collected at the end of the gastrointestinal digestion were briefly vortexed with 5 mL of chloroform, followed by a 10 min centrifugation (Sigma 4K15, Roedermark, Germany) at 343× *g*, at room temperature. The bottom chloroform layer was collected and set aside, and the extraction procedure was repeated with the top layer of the centrifuged mixture. The bottom chloroform layer of the second extraction and centrifugation was added to the first chloroform layer, mixed, and analyzed in a UV/VIS spectrophotometer (Jasco V560, Easton, PA, USA) at 760 nm (absorbance peak). The concentration of PC was determined by interpolating the absorbance of the samples to an analytical curve constructed with a standard solution of gallic acid, as described in Section 2.3.

### 2.7. Statistical Analyses

Statistical analyses were performed using the analysis of variance (ANOVA) procedure with STATISTICA™ v7.0 (Statsoft, Inc., Tulsa, OK, USA) software for Windows. Tukey’s test was applied to detect differences of means, and *p* < 0.05 was considered statistically significant.

For nonlinear regression analysis, Equation (6) was fitted to the data with nonlinear regression, using a package of STATISTICA™ v7.0 (Statsoft, Inc., Tulsa, OK, USA). The Levenberg–Marquardt algorithm for the least squares function minimization was used. The quality of the regressions was evaluated on the basis of the determination coefficient, R^2^, the root-mean-square error (RMSE, i.e., the square root of the sum of the squared residues (SSE) divided by the regression degrees of freedom) and residual visual inspection for randomness and normality. R^2^ and SSE were obtained directly from the software. The precision of the estimated parameters was evaluated using the standardized halved width (SHW%), which was defined as the ratio between the 95% standard error (obtained from the software) and the value of the estimate.

## 3. Results and Discussion

### 3.1. Particle Size and Morphology

**Figure 1:**
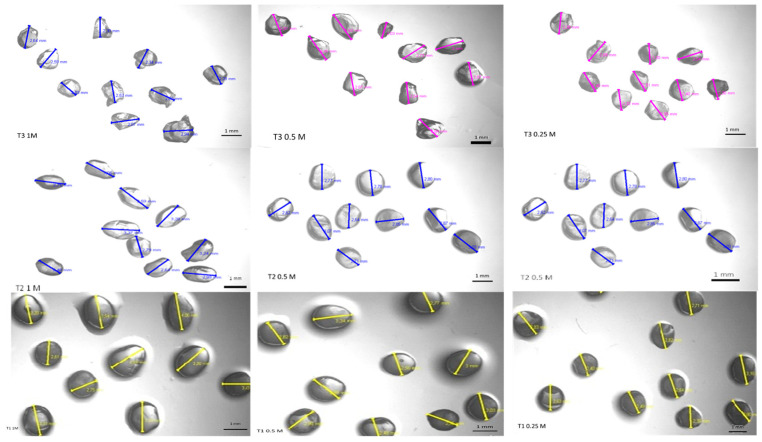
Size and morphology of the alginate particles produced with different sodium alginate and calcium chloride concentrations visualized through a microscope (magnification of 40×). T1, T2, and T3 contained 2%, 1.5%, and 1% of sodium alginate, respectively, and were crosslinked with calcium chloride at concentrations of 1, 0.5, and 0.25 mol·L^−1^. All alginate solutions contained phenolic compounds (2.55 mg gallic acid·g^−1^ of microalgae).

Figure 1 shows the morphological characterization of the alginate particles, produced with different concentrations of sodium alginate and calcium chloride, which were performed using an optical microscope at 40× magnification.

The images show that the particles obtained from the different treatments were morphologically similar, with a spherical or oval shape, but some showed a small salience due to the dripping process. Another relevant factor is that the particles became more spherical for higher concentrations of sodium alginate, which may be related to the higher viscosity of the solution. The particles are left in contact with the solution for crosslinking for a short period of time, usually a few minutes, before being washed with distilled water. The crosslinking occurs through the formation of a complex coacervate membrane when an alginate solution is dripped directly into a calcium chloride solution. In the study of Taofiq et al. [50], the morphology of alginate microspheres was also verified through optical microscopy, and the authors observed a consistent spherical morphology with particles of various sizes showing no agglomeration. In another study, aqueous extracts of stevia leaves and Aronia pomace rich in bioactive compounds were encapsulated in calcium alginate particles using an ionic gelling method, to be used as a green insecticide [51]. The authors observed through an optical microscope that the produced particles were almost spherical; however, after drying to a constant mass, their sphericity was lost, i.e., the surface of the dried particles was no longer smooth and rounded. This was explained by the collapse of the water–gel network during the drying process [51,52].

It is known that particle size is influenced by the solution viscosity, homogenization, time, needle diameter, and distance to the settling solution (CaCl_2_) [53,54]. From Table 1, it can be seen that sodium alginate concentration directly influences the particle size, with solutions with higher concentrations of alginate showing higher viscosity [55], while producing larger particles under the same remaining conditions.

Similar results have been obtained by other authors. Bassani [56] obtained alginate spheres of uniform size with an average diameter of 3.52 mm. Laos et al. [57] studied the encapsulation of sea buckthorn juice (*Hippophaerhamnoides* L.), rich in β-carotene, using an ionotropic gelation process (furcellaran particles). In this case, the size of the particles was influenced by the nature and concentration of the cation, by the polymer, and by the proportion of juice and furcellaran used. The size was evaluated with the aid of a micrometer, and it was found that the larger-diameter particles (4.49 to 4.65 mm) were those with the highest concentration of furcellaran. Other authors encapsulated anthocyanins of hibiscus extract by ionic gelling and of urucum extract by internal gelation, and they obtained particle sizes of 2 mm [58] and 0.697 mm [19], respectively.

Another relevant factor is the effect of different concentrations of CaCl_2_ (Figure 1, Table 1), which is one of the most effective gelling agents since it establishes the link between the alginate chains through ionic interactions. The structure formed has the capacity to retain water in the polymeric structure, which provides greater stability, as it has a higher mechanical resistance than other salts also studied for encapsulation, with the gelling speed being directly proportional to the calcium concentration [59,60]. Thus, the immobilization technique in alginate occurs via the reaction of the carboxylic groups present in adjacent chains of alginate polymer with polyvalent cations, such as calcium chloride, for the formation of a three-dimensional network, resulting in the crosslinking of sodium alginate with Ca^2+^ ions, which assists in the protection of phenolics [61].

### 3.2. Encapsulation Efficiency (%)

**Figure 2:**
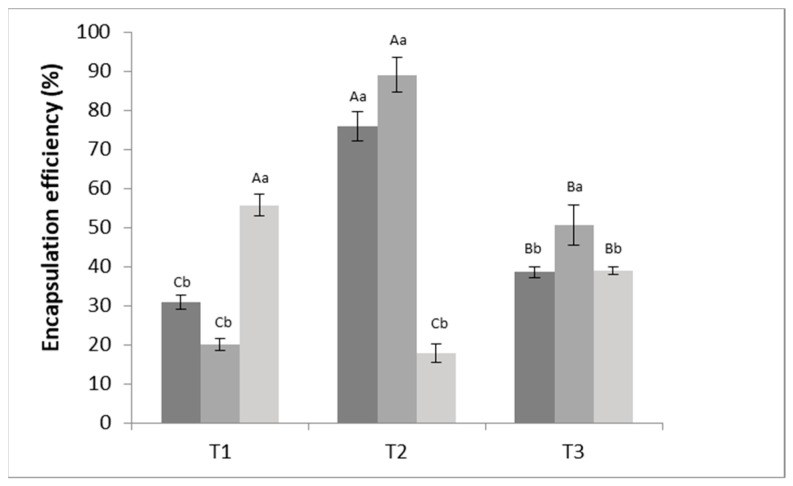
Encapsulation efficiency (%) of phenolic compounds incorporated in alginate particles produced using different alginate and CaCl_2_ concentrations. T1 = 2% sodium alginate; T2 = 1.5% sodium alginate; T3 = 1% of sodium alginate. Different colors of the bars represent different calcium chloride concentrations (■—1 mol·L^−1^, ■—0.5 mol·L^−1^, ■—0.25 mol·L^−1^). Averages followed by uppercase letters are related to significant differences among tests T1, T2, and T3, while lowercase letters are correlated to significant differences according to molar concentrations within the same test (*p* ≤ 0.5).

The encapsulation efficiency (*EE*) defines the quantity of the bioactive compound retained within the particles and depends, among other factors, on the affinity between the wall material and the encapsulated compound [62]. From Figure 2, it can be observed that the concentration of polymer used (i.e., alginate) significantly (*p* < 0.05) affected *EE*.

The particle formulation that exhibited the higher *EE* was T2—0.5 mol·L^−1^, which was composed of 1.5% sodium alginate and 0.5 mol·L^−1^ CaCl_2_. In fact, the ionic gelation technique was revealed to be highly efficient, encapsulating around 88.97% of PC into the alginate particles. Therefore, it was observed that emulsification/internal ionic gelation is a relevant and adequate technique to produce sodium alginate particles encapsulating PC. Furthermore, it can be stated that alginate is an excellent matrix for encapsulating PC extracted from *Spirulina* sp. LEB-18. Given these results, the formulation T2 with 0.5 mol·L^−1^ of CaCl_2_ was chosen to proceed with the tests. For an adequate alginate concentration, an increase in the amount of calcium ions produces gels with time-independent properties, and more calcium additions form a permanent gel structure [63]. Thus, calcium ions react with alginate, establishing crosslinks with its molecules. The mechanism of gel formation is based on the interaction between alginate molecules and calcium ions, which is explained by the “egg box” model, where calcium ions fit between the guluronic acid chains of alginate identically to an egg inside an egg box [55,64,65].

It is interesting that, in the present study, a higher *EE* was obtained with intermediate concentrations of both alginate and sodium chloride. Some works have shown that, depending on the combination of experimental parameters, the use of a lower sodium alginate concentration results in lower *EE* [66,67]. This behavior can be explained by the fact that a higher alginate concentration increased the viscosity of the solution and would consequently lead to the entrapment of higher amounts of the bioactive compound, which partially explain the results of the present work. As for the influence of calcium chloride, it has been shown, in other studies, that a higher concentration of calcium chloride led to lower EE [66]. This could be explained by the fact that calcium ions can bind with the limited carboxylic groups of alginate, leading to the formation of a strong thermostable gel. Once the lead sites are fully occupied, excess calcium ions would no longer be able to incorporate the bioactive compound [66]. This is probably the reason why, in the present work, the higher concentration of calcium chloride tested decreased the *EE.*

Other studies have also stated that *EE* of PC in the hydrogel microbeads can be affected by phenolic loss from the droplet to the hardening solution prior to gelation or from hydrogel particles during hardening, until an equilibrium is established between PC inside the hydrogel particles and the surrounding solution [26].

When compared with other works, the value found (88.97%) in this work for the T2—0.5 mol·L^−1^ formulation is typically higher than those reported. For example, Belščak-Cvitanović et al. [68] found an average total polyphenol *EE* of up to 73% in alginate particles. In another study, Li et al. [69] investigated the encapsulation of tea polyphenols (TP), and their results indicated that the *EE* values of TP in plain alginate granules, as well as granules reinforced with inulin, gum Arabic, and chitosan, were 38.51%, 36.48%, 48.56%, and 57.76%, respectively, which are lower than the value found in the present study. On the other hand, Yilmaztekin et al. [70] found a higher encapsulation efficiency for the encapsulation of peppermint essential oil (98.4% ± 4.3%) in Ca–alginate particles. Other authors encapsulated urucum extract using external gelation and internal gelation techniques and concluded that calcium alginate beads are suitable for the encapsulation of large molecules, especially hydrophobic materials, obtaining encapsulation efficiencies greater than 90% [19,71]. Flamminii et al. [72] developed alginate-based microparticles formulated with different polymers (alginate, alginate–pectin, alginate–whey protein isolate, and alginate–caseinate sodium) and enriched with phenolic extract from olive leaves, and they found that the *EE* of the phenolic extract was higher for the matrix composed by of alginate alone (83%), demonstrating that alginate particles are suitable for the incorporation of phenolic extracts.

### 3.3. Swelling Degree

The swelling degree (*S*) of the alginate particles with phenolic extract from *Spirulina* sp. LEB-18 was used in this study to determine the hydration level of the particles at a given time. The results obtained at times (*t*) 0, 30, 60, 90, and 120 min show that the particles maintained the swelling (*S*) (*S*t_0_ = 30.3 ± 0.28; *S*t_30_ = 36.75 ± 0.35; *S*t_60_ = 36.75 ± 0.35; *S*t_90_ = 40.92 ± 0.95; *S*t_120_ = 50.15 ± 0.42), with a gradual moisture gain in relation to the initial mass throughout the test. Thus, it was appropriate to set the time required for maximum *S* at 120 min. The determination of *S* of the particles is an important factor, since it can help to understand the release mechanisms of the bioactive compounds. When the particles are exposed to continuous water absorption, the gel particles erode due to excessive swelling. Factors such as the crosslinking degree, hydrophilicity, and hydrophobicity of the polymer influence the value of *S* [73,74].

### 3.4. Release Profile

**Figure 3:**
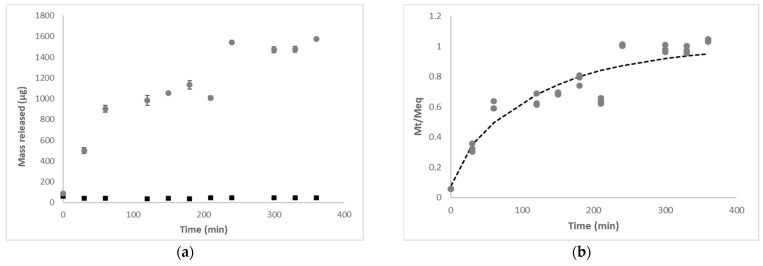
Profile of phenolic compounds release from alginate particles (T2—0.5 mol·L^−1^ formulation, 1.5% alginate and 0.5 mol·L^−1^ CaCl_2_): (**a**) mass of phenolic compounds released at pH 1.5 (■) and pH 7.2 (●); (**b**) experimental data of phenolic compounds release at pH 7.2 (●) and description of linear superimposition model (*i* = 1) (--).

It is known that the release of a bioactive compound from a polymeric matrix may often be caused by some external environmental factors such as pH, ionic strength, solvent composition, buffer composition, temperature, or pressure [75]. The release of the PC from the alginate particles was evaluated at two different pH values (Figure 3). The release profile of PC in an acidic medium (pH 1.5) simulated the gastric pH conditions, while the release profile in a neutral pH medium (pH 7.2) simulated the intestinal pH conditions.

As shown in Figure 3a, in an acidic medium, the release of the PC was not observed, while a controlled release occurred at neutral pH, which can be explained by the high solubility of the alginate at this pH. It is known that the presence of carboxyl groups that dissociate or incorporate protons (H^+^) causes the alginate behavior to be pH-dependent. With the pH below the pKa range of the constituent acids (pKa of the β-D-mannuronic = 3.38, pKa of α-L-guluronic = 3.65), the polymer incorporates protons and becomes insoluble, protecting the compound encapsulated. Above this value, the alginate becomes soluble, releasing the active compound [76,77].

It was also observed that the particles swell and start to disintegrate at pH 7.2 over time, thus allowing the release of the active compound as disintegration occurs (not shown). Moreover, it is known that alginate gels degrade and precipitate in 0.1 mol·L^−1^ phosphate buffer at pH 7.2 because the calcium ions are converted into calcium phosphate, leading to a rapid release of the PC. Phosphate ions in PBS lead to a fast dissolution of the alginate materials; thus, water uptake may be distorted due to fast calcium leakage [78]. Similarly, alginate gels show a high porosity at this pH, resulting in high diffusion rates. The structure becomes more flexible, leading to relaxation of the chains. The polar groups are then hydrated and swell the polymeric network, forming a hydrogel. The water that fills the spaces between the chains promotes a favorable environment for the dissolution and transport of the compound [79,80].

It is known that the release of the bioactive compound from a polymeric matrix may be governed by different release mechanisms such as diffusion, erosion/disintegration, swelling, or a combination thereof [81]. In order to evaluate mechanisms involved in PC release from alginate particles at pH 7.2 other than disintegration, the experimental results were analyzed by fitting the LSM (Equation (6)) (Figure 3). Table 2 presents the regression analysis results of the LSM fitting, showing that this model adequately described the experimental data with relatively good regression quality (R^2^ ≈ 0.90), and that some parameters were estimated with good precision.

The parameter *X* is defined as the Fick’s diffusion contribution to the total release from the alginate particles (*M_∞,F_/M_t_*); from Table 2, it can be observed that *X* < 0.5, which indicates that, at pH 7.2, relaxation, driven by the swelling ability of the polymer, is the governing phenomenon, which is in accordance with the swelling degree results that previously showed that alginate particles present high swelling ability. Furthermore, although the relaxation rate constant (*k_R_*) was lower than the Fickian rate constant (*k_F_*) (Table 2), these results must be analyzed with care, given the low estimate precision associated with the *k_F_*. In systems where the release mechanism is influenced by swelling, the polymer initially swells by absorption of the liquid medium, undergoing a transition from a glassy state to a gelatinous state by interaction with the solvent. The polymer chains in the gelatinous state are more mobile than in the glassy state, allowing the encapsulated ingredient to diffuse rapidly through the polymer matrix.

### 3.5. Differential Scanning Calorimetric Analysis (DSC)

Differential scanning calorimetry (DSC) is an analytical tool routinely used to characterize the state of alginate particles [82]. The ionic gelation involves the transition of the liquid material to a gel [83,84,85]. Using DSC it was possible to observe an exothermic event for both particles (alginate particles without PC (control particles) and alginate particles with PC). For the control particles, the behavior exothermic event occurred at an initial temperature of 95.33 °C and a final temperature of 205.33 °C. As for the particles containing PC, this exothermic conduct occurred from an initial temperature of 121.21 °C to a final temperature of 207.83 °C (Table 3).

The exothermic peak resulted from the reactions involved in alginate degradation, as endothermic peaks are related to water loss associated with the hydrophilic groups of the alginate [86,87,88]. The shape of the melting peak of the alginate particles (without PC) (exothermic peak) suggests that it is unstable, because two events occur (dual band) for both studied particles, possibly followed by a change in the material, which, in turn, is indicated by an intense exothermic peak, after particle fusion (results not shown). As for the particles containing PC, the inverse behavior was observed, with a release of the PC, as indicated by the exothermic peaks, which suggests its degradation during thermal analysis.

### 3.6. In Vitro Digestion

In order to be bioaccessible, the compound of interest must be released from the particles during the gastrointestinal digestion; therefore, it is of utmost importance to evaluate the behavior of the alginate particles during the digestion [89,90,91,92]. Throughout digestion, food passes through two main processes (occurring simultaneously), the mechanical movements (i.e., peristalsis), which reduce the particle diameter of food, and enzymatic action, in which macromolecules are hydrolyzed to form more basic and smaller structures, easier to be subsequently absorbed into the bloodstream [93,94,95,96]. Digestion kinetics depend on the physical and chemical characteristics of food and their interaction with the substances involved in the process [95].

Aiming to evaluate the behavior of alginate particles containing PC under gastrointestinal conditions, a static harmonized in vitro model was used to simulate, as close as possible, the human gastrointestinal tract. PC bioavailability can be restricted by its enzymatic degradation before reaching the intestine, which is the highest absorption region. However, alginate particles containing PC can be protected from the harsh stomach environment. Therefore, the behavior of alginate particles with PC was tracked throughout digestion, by determining the total phenolics using the Folin–Ciocâlteu method, the antioxidant effect of the PC using DPPH (Table 4), and the PC bioaccessibility.

After ingestion, the encapsulated PC underwent a series of complex physicochemical and physiological processes (i.e., pH changes, presence of enzymes, and complex forces) in the gastrointestinal system, which could lead to a change in their structure, thus reducing its effectiveness. Table 4 shows that, after the oral phase, there was a slight decrease in the PC concentration for both free and encapsulated samples. After the gastric phase, free and encapsulated PC presented a different behavior; there was a significant decrease in the PC concentration for the free PC sample, whereas the concentration of encapsulated PC was maintained. These results show that the particles could protect the PC from the harsh conditions found in the gastric phase (i.e., high acidic pH, and high ionic strength). Under intestinal conditions, the concentration of PC decreased for both samples, presenting final values of 3.92 ± 0.36 and 9.43 ± 1.86 µg sample/mL for free and encapsulated PC, respectively. As seen from the in vitro release results, the release of PC from alginate particles was significantly reduced at low pH (gastric environment), i.e., in the gastric fluid, the hydrated alginate was converted into a “skin” of alginic acid, which is porous and insoluble. When it transits into the intestine phase, where the pH is higher, the “skin” of alginic acid is converted into a soluble viscous layer, releasing the particles’ content [97,98].

In the literature, there is little information about the maintenance of the antioxidant activity of PC during the digestion process. Therefore, the purpose of this study was also to evaluate the antioxidant activity of encapsulated PC after the simulation of digestion and compare it with that of the non-encapsulated PC. Therefore, the antioxidant activity was measured along the digestion process to evaluate the protective effect of the developed alginate particles, as shown in Table 4. Both the free and the encapsulated PC maintained their antioxidant activity after the oral phase. In the later stages of digestion (i.e., gastric and intestinal phases), there was a decrease in the antioxidant activity of the PC encapsulated in the particles; however, this decrease was lower when compared to the free PC, justifying the use of alginate particles as the encapsulating matrix. Therefore, it can be concluded that the encapsulation of the PC in alginate particles decreased their loss during digestion and can be used as a strategy to maintain their stability. In the study by Davidov-Pardo et al. [99], with 23 commercial vegetable juices, it was observed that the value of DPPH increased after gastric digestion, but was slightly reduced after intestinal digestion, which is in agreement with the results of the present study.

Other authors encapsulated kenaf seed oil using pectin and alginate with chitosan coating and evaluated the changes in antioxidant activity and bioactive properties before and after in vitro digestion when compared with the non-encapsulated oil. The authors concluded that the microencapsulation of kenaf seed oil offered a controlled and effective release system compared to the oil without encapsulation [100].

The bioavailability of a compound that is orally ingested consists of the fraction of the compound that is absorbed after solubilization in the gut and is available for physiological functions (i.e., reaches the systemic circulation in an active form). Before bioavailability studies, it is common to assess bioaccessibility, which is defined as the fraction of a compound that is released from the matrix in the gastrointestinal tract and is available for absorption. Thus, bioaccessibility can be considered an indicative value of the maximum oral bioavailability [101]. Bioaccessibility was assessed at the end of simulated intestinal digestion for free PC and PC encapsulated in alginate particles. Results showed a high increase in bioaccessibility values when the PC was encapsulated in alginate particles: 32.24% ± 3.50% for free PC and 61.76% ± 8.80% for PC encapsulated in alginate particles. The bioaccessibility values are in agreement with the values obtained for other encapsulation systems loaded with resveratrol (a type of polyphenol), where bioaccessibility was shown to increase from 60% to 73% for resveratrol in free form and encapsulated in biopolymer particles, respectively [102]. In another study, Machado et al. [103] observed an increase in the bioaccessibility of PC from 31.65% (free form) to 35.83% and 45.89% when the PC were encapsulated in different liposomes.

## 4. Conclusions

This study demonstrated the possibility of preparing alginate particles containing phenolic extract of *Spirulina* sp. LEB-18 with high encapsulating efficiency. These particles also showed potential to act as a controlled delivery system of phenolic compounds, and it was found that their release is pH-dependent. Furthermore, alginate particles were effective in protecting the compounds during digestion, once more phenolic compounds and increased antioxidant activity were observed throughout the digestion when compared to the free phenolic compounds, and the bioaccessibility of the phenolic compounds was increased almost twofold after its encapsulation in alginate particles. Once the technique used for the encapsulation of the phenolic compounds (i.e., ionic gelation technique) presents several advantages such as practical implementation, easy scale-up, and the use of biocompatible materials, alginate particles represent a viable and cost-effective alternative for the encapsulation of phenolic extracts for future application in foods.

## Figures and Tables

**Table 1:** Size of particles obtained using different concentrations of sodium alginate and calcium chloride (CaCl_2_).

Alginate Concentration (%)	Particle Size (mm)		
	CaCl_2_ Concentration (mol·L^−1^)		
	1.0	0.5	0.25
2	3.33 ± 0.42 ^A,a^	2.91 ± 0.28 ^A,a,b^	2.64 ± 0.26 ^A,B,b,c^
1.5	3.02 ± 0.35 ^A,B,a,b^	2.81 ± 0.15 ^A,a,b^	2.49 ± 0.27 ^B,b,c^
1	2.83 ± 0.22 ^B,a^	2.80 ± 0.43 ^A,a^	2.87 ± 0.35 ^A,a^

Different uppercase letters in the same column indicate statistical differences between the samples at *p* ≤ 0.05. Different lowercase letters in the same row indicates a statistical difference.

**Table 2:** Results of fitting the linear superimposition model (LSM) (*i* = 1) to experimental data of phenolic compounds release at pH 7.2. Evaluation of the quality of the regression on the basis of RMSE and R^2^. The precision of estimates was evaluated using the SHW% (in parenthesis).

pH	RMSE	R^2^	*X*	*k_F_* (min^−1^)	*k_R_* (min^−1^)
7.2	0.099	0.900	0.204 (110.73%)	7.18 × 10^−2^ (594.49%)	7.63 × 10^−3^ (28.43%)

RMSE: root-mean-square error; R^2^: quality of the regression; *X*: fraction of compound released by Fickian transport; *k_F_*: Fickian diffusion rate constant; *k_R_*: relaxation rate constant.

**Table 3:** Thermal properties of alginate particles with and without phenolic extract of *Spirulina* sp. LEB-18.

Sample	Thermal Properties		
	*T_o_* (°C)	*T_m_* (°C)	*T_f_* (°C)
Alginate particles	95.33	142.33	205.33
Alginate particles with phenolic compounds	121.21	172.5	207.83

*To*: onset temperature; *T_m_*: transition phase temperature; *T_f_*: final temperature.

**Table 4:** Quantification of phenolic compounds and antioxidant activity of free phenolic compounds and alginate particles with phenolic compounds submitted to gastrointestinal digestion.

Quantification of Phenolic Compounds (µg _sample_/mL)				
Sample	Initial	Oral Phase	Gastric Phase	Intestinal Phase
**Phenolic compounds**	11.29 ± 1.79 ^A;a^	9.69 ± 0.20 ^A;c^	6.63 ± 0.26 ^B;e^	3.92 ± 0.36 ^C;f^
**Alginate particles with phenolic compounds**	13.87 ± 1.22 ^D;a^	12.29 ± 1.45 ^D;E;b^	12.21 ± 0.68 ^D;E, d^	9.43 ± 1.86 ^E;f^
Antioxidant activity—%ASR *				
	**Initial**	**Oral phase**	**Gastric phase**	**Intestinal phase**
**Phenolic compounds**	15.27 ± 1.40 ^A;b^	17.14 ± 2.22 ^A;d^	19.79 ± 2.16 ^A;f^	2.88 ± 0.41 ^B;h^
**Alginate particles with phenolic compoundst**	30.16 ± 4.72 ^C;a^	35.99 ± 2.80 ^C;c^	21.66 ± 2.50 ^D; e^	12.14 ± 3.09 ^E;g^

* ASR = scavenging activity of radicals; different uppercase letters in the same row indicate a statistical difference, while lowercase letters in the same column indicate no difference between samples (*p* < 0.05).

## Data Availability

Not applicable.
